# RxMap: an LLM-assisted tool for medication normalization

**DOI:** 10.1093/jamiaopen/ooag085

**Published:** 2026-05-30

**Authors:** Eero Korpela, Leah H Rubin, Raha M Dastgheyb, Yanxun Xu

**Affiliations:** Department of Applied Mathematics and Statistics, Johns Hopkins University, Baltimore, MD, United States; Departments of Neurology, Johns Hopkins University School of Medicine, Baltimore, MD, United States; Department of Psychiatry and Behavioral Sciences, Johns Hopkins University School of Medicine, Baltimore, MD, United States; Department of Molecular and Comparative Pathobiology, Johns Hopkins University School of Medicine, Baltimore, MD, United States; Department of Epidemiology, Johns Hopkins University Bloomberg School of Public Health, Baltimore, MD, United States; Departments of Neurology, Johns Hopkins University School of Medicine, Baltimore, MD, United States; Department of Applied Mathematics and Statistics, Johns Hopkins University, Baltimore, MD, United States; Division of Quantitative Sciences, Sidney Kimmel Comprehensive Cancer Center, Johns Hopkins University School of Medicine, Baltimore, MD, United States

**Keywords:** medical concept normalization, RxNorm, large language models, data harmonization

## Abstract

**Objective:**

To develop a freely available, researcher-oriented system for accurate normalization of free-text medication strings to standardized RxNorm ingredient-level concepts.

**Methods:**

RxMap implements a fully automated normalization pipeline that combines deterministic RxNorm candidate generation with large language model (LLM)–assisted lexical parsing and hierarchical ingredient-level reconciliation. Raw medication strings are normalized to RxNorm ingredient-level (IN/MIN) concepts. ATC codes are assigned post-normalization, and an optional review interface supports transparent human review.

**Results:**

Evaluation on 22 624 unique medication strings from the IPUMS MEPS dataset demonstrated substantial improvements over deterministic RxNorm matching alone. The best-performing RxMap configuration improved RxCUI-level precision, recall, and F1-score from 0.865, 0.853, and 0.859 to 0.969, 0.964, and 0.966, respectively, with similar gains at the ingredient level.

**Conclusion:**

RxMap provides accurate, scalable normalization of free-text medication data to RxNorm concepts. The system operates fully automatically, offers optional review for quality control, and enables reproducible batch processing for research workflows.

## Introduction

The normalization of medication data to standardized drug terminologies is foundational for transforming heterogeneous healthcare data into analyzable and interoperable resources.^1^

Without normalization, medication information recorded across disparate sources cannot be reliably aggregated, compared, or analyzed at scale. In real-world settings, including electronic health records, surveys, dispensing systems, and clinical databases, medication information is recorded using highly heterogeneous conventions reflecting local practices and data collection contexts.^2–5^ These entries frequently combine brand and generic names, abbreviations, misspellings, truncated strings, and inconsistent expressions of strength, units, and multi-ingredient formulations. Aligning such inputs to standardized drug concepts is essential for reproducible research, cross-study comparison, and downstream analytics. Despite this need, medication normalization in many research workflows remains partially manual, making it slow, difficult to scale, and error-prone.^6^^,^^7^

Standardized terminologies such as RxNorm and the Anatomical Therapeutic Chemical (ATC) classification system provide the semantic backbone for addressing this challenge. RxNorm offers a comprehensive, ingredient-centric vocabulary widely used in the United States to represent clinical drugs and their relationships, enabling normalization of heterogeneous medication strings to a common identifier space.^8^ ATC annotates medications into therapeutic and pharmacologic classes, supporting downstream analyses such as drug utilization, similarity, and class-level comparisons.^9^ Together, these resources enable medication data to be represented in consistent and analytically useful forms. However, the practical task of mapping messy, real-world medication strings to these standards remains difficult.

This work focuses on the task of medication normalization, defined as mapping free-text medication name strings to standardized drug concepts. We assume medication strings are already available from sources such as surveys or dispensing records and seek to normalize these inputs to RxNorm ingredient-level (IN) or multi-ingredient (MIN) concepts. Where relevant for downstream analysis, normalized concepts are further annotated with ATC classes, with explicit handling of complex combination products.

Existing computational approaches to medication normalization include lexicon- and rule-based matching against RxNorm, fuzzy string retrieval, and embedding-based methods that retrieve and rank candidate concepts.^10–12^ More recently, large language models (LLMs) have been explored for abbreviation expansion and candidate generation from free-text inputs.^13^^,^^14^ While these approaches improve coverage, several challenges persist for messy, non-clinical medication strings: brittleness to misspellings and truncated forms; difficulty selecting among many plausible candidates with overlapping attributes; uneven support for multi-ingredient formulations; and limited integration of ATC annotation within open, reproducible workflows. Widely used clinical NLP toolkits (eg, MedXN, MedEx, cTAKES) perform well at extracting medications from clinical narratives but are not designed for robust normalization, and comprehensive ATC integration remains uncommon.^11^^,^^15–17^

To address these gaps, we present RxMap, a free, researcher-oriented web application and pipeline for medication normalization with optional ATC annotation (https://www.rx-map.com/). RxMap combines deterministic RxNorm candidate generation with constrained LLM-assisted lexical parsing and resolves mappings at the RxNorm IN and MIN levels. The system explicitly supports complex combination products and integrates a RxNorm-to-ATC crosswalk to enable class-level analyses. An interactive review interface provides optional inspection and correction of automatically generated mappings and supports reproducible batch-processing workflows. Unlike proprietary normalization services embedded within specific electronic health record ecosystems,^18^ RxMap is designed as an open, standalone tool for research use.

## Methods

### Overview

**Figure 1.**
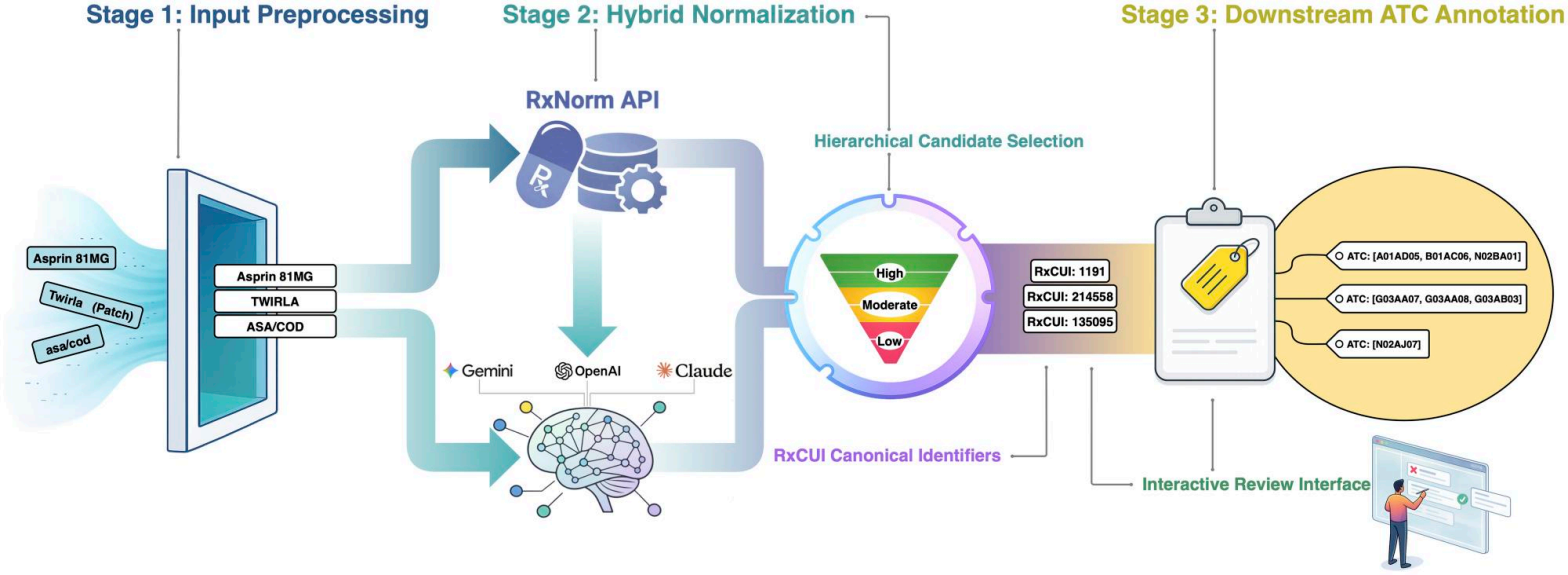
Overview of RxMap pipeline. The pipeline integrates preprocessing, deterministic and LLM-assisted candidate generation, hierarchical candidate selection to RxNorm concepts, and automated ATC code annotation. Cached mappings and a stored database support reproducibility, while a frontend review interface allows for manual review and resolves ambiguous cases before producing finalized mappings for export.

RxMap implements a fully automated pipeline for normalizing raw medication strings to standardized RxNorm ingredient and multi-ingredient level concepts (IN/MIN), represented by RxNorm concept unique identifiers (RxCUIs). The pipeline comprises three stages: (i) input preprocessing, (ii) ingredient-level normalization via deterministic RxNorm matching augmented with constrained LLM-assisted parsing and hierarchical candidate selection, and (iii) downstream pharmacologic annotation using ATC classifications (Figure 1).

### Input preprocessing

Raw medication strings undergo minimal preprocessing to inputs for consistent querying and caching, including removal of parenthetical content, whitespace normalization, and uppercasing. No spelling correction, abbreviation expansion, or drug-specific heuristics are applied, preserving the original semantics for downstream matching.

### Hybrid normalization

For each preprocessed medication string, RxMap generates candidate ingredient-level representations from two sources and reconciles them using a hierarchical selection strategy. A deterministic candidate is obtained via RxNorm’s approximate string-matching algorithm (getApproximateMatch function as described in Peters et al.^10^) which performs fuzzy lexical matching based on Jaccard similarity scores between normalized token sets, and is then resolved by default paths between related concepts within RxNorm to an active generic concept (IN or MIN). After deterministic mapping, an LLM parses the raw string to produce a standardized list of generic active ingredient names. When a deterministic candidate is available, it is provided in the LLM prompt as supplementary context; however, the LLM is explicitly instructed to prioritize the raw medication string and to disregard the deterministic candidate if inconsistencies arise. These two sources provide complementary candidate ingredient sets. Further details on deterministic and LLM-assisted candidate generation are provided in Supplementary Materials S2.

Candidate ingredient sets are reconciled by first accepting exact agreement when available. We denote this perfect match between ingredient sets as a high confidence match. When lexical discrepancies occur between the two ingredient sets (eg, {ACETYLSALICYLIC ACID, CODEINE} vs {ASPIRIN, CODEINE PHOSPHATE}), both sets are normalized to canonical RxNorm ingredient names by independently applying the deterministic mapping procedure described above to each ingredient string. After normalization, both sets resolve to the same canonical representation (eg, {ASPIRIN, CODEINE}), and the sets are re-compared for exact equivalence. Equivalent sets after normalization constitute a normalized perfect match and are labeled as moderate confidence within RxMap.

**Figure 2.**
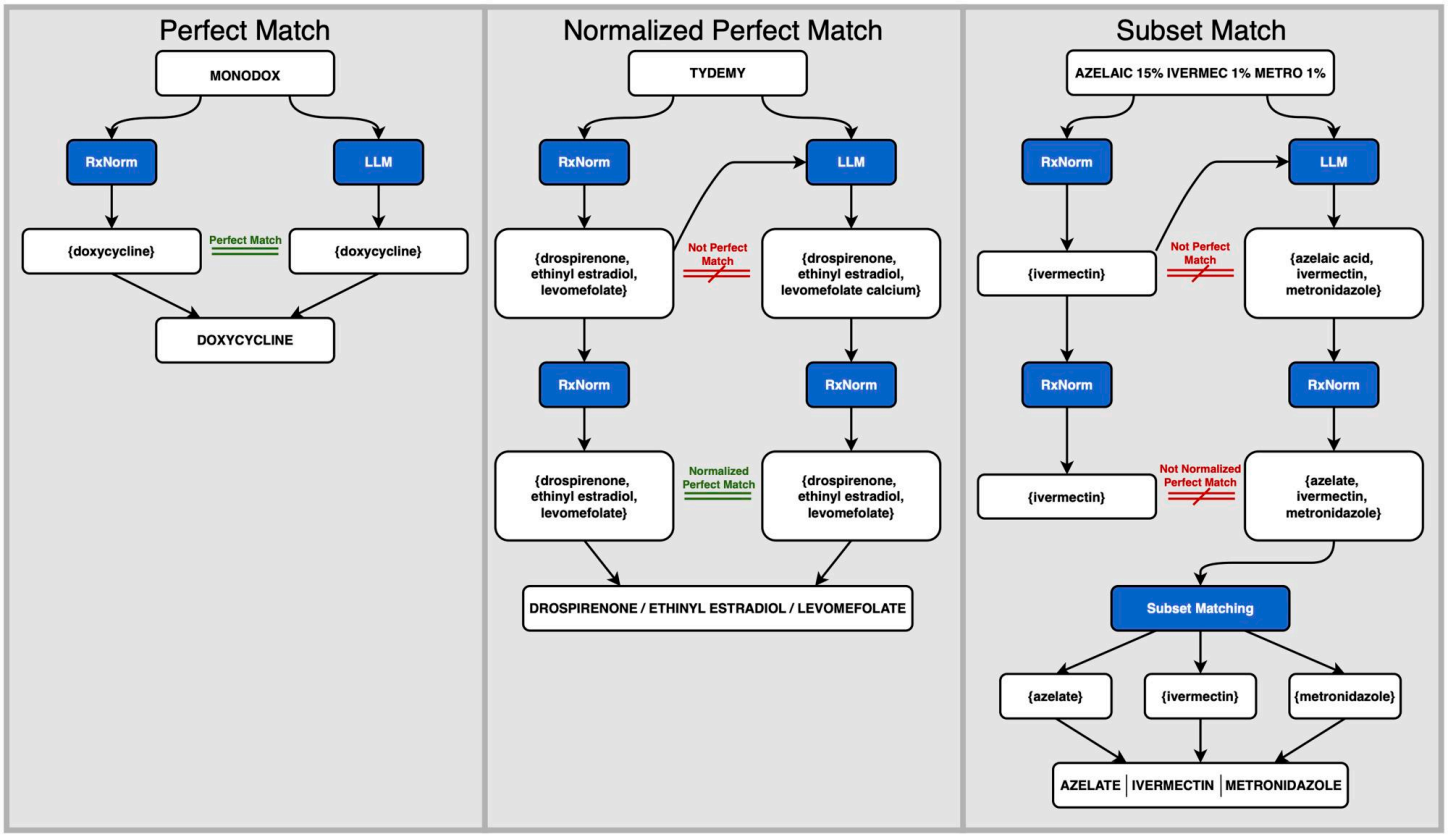
Hierarchical candidate selection logic for three medication concept normalization pathways in RxMap: Perfect Match (high confidence match), Normalized Perfect Match (moderate confidence match), and Subset Match (low confidence match).

If agreement is still not achieved, subset matching is applied to resolve combination products in a structure-preserving manner. Specifically, the LLM-derived ingredient set is decomposed into candidate subsets evaluated in descending order of size. Each subset is deterministically matched to valid IN/MIN concepts when available. When a valid subset with exact ingredient match is identified, it is selected as a finalized mapping and its constituent ingredients are removed from the remaining set. This process continues iteratively until no further valid subsets can be identified. The final output may therefore consist of multiple RxNorm concepts (eg, a multi-ingredient combination plus one or more single ingredients) that collectively cover the LLM-derived ingredient set. Because subset matching operates exclusively on the LLM-generated set without deterministic reconciliation, these matches are designated as low confidence. Examples for the three mapping outcomes (high, moderate, and low confidence) are shown in Figure 2.

As defined above, mapping confidence is a structured, rule-based categorization based on match type intended to support user interpretability and workflow efficiency in RxMap, rather than a computed probabilistic score. Empirical verification of these categories is provided in Supplementary Materials S2.2.1.

### ATC classification (post-normalization)

ATC classification is applied as a post-normalization annotation step on finalized RxNorm concepts. ATC codes are preferentially obtained directly from RxNorm when available; otherwise, RxClass is queried.^19^ Assignments from RxClass are accepted only when the linked concepts share identical active ingredient sets with the normalized RxNorm concept, ensuring pharmacologic consistency. When combination-level ATC codes are unavailable, ATC annotations may be provided for classifiable subsets and/or individual ingredients and labeled as partial coverage. Implementation details of ATC assignment, ingredient-level validation, and partial coverage handling are provided in Supplementary Materials S3.

### Batch processing, review interface, and reproducibility

RxMap supports single-string queries and batch CSV uploads. In batch mode, each input medication string is represented as a single row with normalized RxNorm concepts displayed as structured annotations. Users can filter by mapping confidence and optionally edit automatically generated mappings prior to export. The system queries the live RxNorm API, uses deterministic decoding for LLM-assisted parsing, and applies caching to support reproducibility. Additional details on the user interface, batch review workflows, and session persistence are provided in Supplementary Materials S4, and system architecture, caching, and reproducibility safeguards are described in Supplementary Materials S1 and S5.

## Results

We evaluated RxMap using medication data from two datasets: IPUM MEPS^20^ and a harmonized dataset denoted CHARTER/NNTC/HNRP.^21–23^ The datasets were comprised of 22,624 and 1,363 unique raw medication strings, respectively. Each dataset was mapped to establish a gold-standard RxCUI and corresponding ingredient set. Dataset details are provided in Supplementary Materials S6. Table 1 provides randomly sampled examples with gold-standard mappings to illustrate the diversity and difficulty of the normalization task.

**Table 1.** Representative examples of free-text medication strings from the IPUMS MEPS and CHARTER/NNTC/HNRP datasets and gold-standard RxNorm mapping.

Dataset	RXNAME	Mapped medication string	Mapped RxCUIs
IPUMS MEPS	PEDIOTIC	HYDROCORTISONE/NEOMYCIN/POLYMYXIN B	217627
	DIPHEN-ATROP	DIPHENHYDRAMINE | ATROPINE	3498 | 1223
	LO/OVRAL 28	ETHINYL ESTRADIOL/LEVONORGESTREL	214558
	DDAVP NASAL SPRAY*5ML	DESMOPRESSIN	3251
	LOESTRIN FE 1/20	ETHINYL ESTRADIOL/NORETHINDRONE | FERROUS FUMARATE	384410 | 24941
	GANI-TUSS-DM NR (A.F., S.F., RASPBERRY)	DEXTROMETHORPHAN/GUAIFENESIN	214488
	POTASS. CL 10MEQ @@	POTASSIUM CHLORIDE	8591
	PPA/PYRIL/P-TLOX/PHENIR	PHENIRAMINE/PHENYLPROPANOLAMINE/PHENYLTOLOXAMINE/PYRILAMINE	689741
	ACETAM CODEINE #3	ACETAMINOPHEN/CODEINE	817579
	RYNATAN/R-TENNATE	CHLORPHENIRAMINE/PHENYLEPHRINE/PYRILAMINE	214416
CHARTER/NNTC/HNRP	ADVIL PM	DIPHENHYDRAMINE/IBUPROFEN	644895
	ACETAMINOPHEN; COMB.; DEXTROMETHORPHAN HBR; DOXYLAMINE SUCCINATE; PSEUDOEPHEDRINE HCL	ACETAMINOPHEN/DEXTROMETHORPHAN/DOXYLAMINE/PSEUDOEPHEDRINE	466524
	NEOMYC/POLYMY/BACITR/LIDO	BACITRACIN/LIDOCAINE/NEOMYCIN/POLYMYXIN B	1007611
	PENICILLIN G, CLEMIZOL	CLEMIZOLPENICILLIN/PENICILLIN G	1007623
	GS-5885 (NS5A INHIBITOR)	LEDIPASVIR	1591922
	DIPHENHYDRAMINE HCL; COMB.; TETRACYCLINE (EENT)	DIPHENHYDRAMINE | TETRACYCLINE	3498 | 10395
	UNIVASC	MOEXIPRIL	30131
	PREMPRO	ESTROGENS, CONJUGATED (USP)/MEDROXYPROGESTERONE	1006917
	MORPHINE HYDROCHLORIDE	MORPHINE	7052
	PYLERA	BISMUTH SUBCITRATE/METRONIDAZOLE/TETRACYCLINE	1007769

Examples illustrate common challenges including brand/generic variation, abbreviations, misspellings, truncation, and multi-ingredient products. For combination drugs, the slash (/) denotes a multi-ingredient RxNorm concept, while the pipe symbol (|) separates distinct drug concepts when a single input string maps to multiple RxCUIs

We assessed the performance of RxMap against two deterministic baselines and examined the effect of two different LLMs backends within the same framework. The four pipelines were: (1) RxNorm-OHDSI, a deterministic baseline developed for the Canada Vigilance database;^24^ (2) RxNorm+, the deterministic matching component of RxMap; (3) RxMap (GPT-5-Nano), the full pipeline using OpenAI’s gpt-5-nano for candidate generation; and (4) RxMap (Gemini-2.0-Flash), the same pipeline using Google’s gemini-2.0-flash. Performance was measured using precision, recall, and F1 at both the RxCUI level (exact match) and ingredient level (constituent identification). Ingredient-level evaluation expands predicted and reference RxCUIs into ingredient sets, enabling partial correctness for multi-ingredient products.

Table 2 presents the comparative performance metrics. The results for the IPUMS dataset demonstrate that the hybrid RxMap pipelines substantially outperform the deterministic baselines in both exact concept matching and ingredient identification. The Gemini-2.0-Flash variant achieved the highest overall scores, with an F1 of 0.966 at the RxCUI level and 0.972 at the ingredient level, representing a major improvement over the RxNorm+ baseline (F1 of 0.859 and 0.874, respectively). The baseline RxNorm-OHDSI pipeline achieved limited recall, primarily due to its inability to robustly map multi-ingredient formulations. RxMap’s standalone deterministic component (RxNorm+) captured a substantial portion of well-formatted entries but frequently failed on inputs with misspellings (eg, “BENZONATE”), truncations (eg, “SULFAMET”), or brand name drugs (eg, “LO-OVRAL-28”). In contrast, the proposed hybrid pipeline addressed these gaps: the LLM component successfully parsed and normalized such challenging strings, and the backend logic preserved the structure of combination products. Performance across the two LLM variants was comparable, with Gemini-2.0-Flash exhibiting modestly better performance, suggesting that RxMap’s performance is robust to the choice of underlying LLM. Similar results for CHARTER/NNTC/HNRP were observed.

**Table 2.** Performance comparison of medication normalization methods on IPUMS MEPS and harmonized CHARTER/NNTC/HNRP dataset.

		RxCUI				Ingredient			
Dataset	Performance Metrics	RxNorm-OHDSI	RxNorm+	RxMap (gpt-5-nano)	RxMap (gemini-2.0-flash)	RxNorm-OHDSI	RxNorm+	RxMap (gpt-5-nano)	RxMap (gemini-2.0-flash)
IPUMS MEPS	F1^a^	0.240	0.859	0.906	0.966	0.300	0.832	0.919	0.972
	Precision^b^	0.244	0.865	0.911	0.969	0.288	0.853	0.929	0.976
	Recall^c^	0.236	0.853	0.900	0.964	0.313	0.812	0.909	0.967
CHARTER/NNTC/HNRP	F1	0.720	0.896	0.919	0.956	0.877	0.898	0.925	0.953
	Precision	0.721	0.896	0.942	0.962	0.890	0.901	0.960	0.966
	Recall	0.718	0.895	0.898	0.950	0.865	0.895	0.892	0.941

a Harmonic mean of precision and recall.

b Proportion of predicted RxCUI/expanded ingredient mappings that are correct.

c Proportion of gold-standard RxCUI/expanded ingredient mappings correctly recovered.

Metrics are reported at the RxCUI level (exact concept match) and the ingredient level (constituent ingredient match) for two deterministic baselines and two variants of the proposed RxMap hybrid pipeline.

## Discussion

This paper presents RxMap, a researcher-oriented system for medication normalization that achieves high accuracy on noisy, real-world medication strings. Across large, heterogeneous evaluation sets, RxMap substantially outperforms deterministic RxNorm matching at both the RxCUI and ingredient levels, demonstrating practical gains in coverage without sacrificing precision.

The observed gains stem from two design choices addressing common medication normalization failures. First, constrained LLM parsing recovers signal from misspellings, abbreviations, truncated brand names, and pharmacy-specific notations that often defeat deterministic methods. Second, hierarchical, ingredient-preserving selection prevents premature collapse of combination products, improving accuracy for multi-ingredient medications common in survey and dispensing data. Together, these components support reliable normalization in realistic settings.

Analysis revealed that most RxMap errors occur during subset matching. Common patterns include resolution to generic ingredient forms rather than specific chemical variants (eg, vitamin D rather than cholecalciferol), mapping herbal supplements to active compounds rather than botanical sources, and selection of incorrect salt forms in branded products. These errors reflect a trade-off: LLM generalization improves robustness to misspellings but can collapse fine-grained chemical distinctions. Low-confidence labeling for subset matches enables users to prioritize review when chemical specificity is critical.

Importantly, RxMap is automation-first: normalization and ATC annotation execute fully automatically at scale, while the review interface is optional and intended for quality control in low-confidence edge cases. This design supports both high-throughput batch processing and targeted auditability when needed, aligning with practical research use where reproducibility and traceability are essential. From a practical standpoint, RxMap introduces modest computational overhead relative to deterministic methods due to LLM inference. In our evaluation, processing averaged ∼3.7 seconds per medication string at ∼$0.0002 per mapping (Gemini-2.0-Flash), supporting feasibility for large-scale batch use. Medication-level caching avoids repeated LLM calls for recurring strings, substantially reducing runtime and cost while preserving reproducibility.

Several limitations warrant further work. Performance depends partly on the underlying LLM, though deterministic validation and caching improve stability. Coverage is limited by RxNorm, leaving rare or newly introduced products challenging. ATC mappings are not always one-to-one, especially for combination therapies, motivating subset-level annotation. The system also focuses on English, U.S.-centric inputs; extending to additional languages, terminologies, and datasets is needed for broader generalizability. A natural extension of this work is integration with pipelines that extract medical concepts from unstructured clinical text. Such integration would enable an end-to-end processing pipeline for transforming unstructured clinical narratives into standardized, analyzable medication data suitable for pharmacoepidemiologic research and clinical decision support applications.

## Supplementary Material

ooag085_Supplementary_Data

## Data Availability

All datasets analyzed in this paper are publicly accessible upon request from https://meps.ipums.org/meps/ and https://hnrp.hivresearch.ucsd.edu/.

## Software availability

RxMap application can be accessed from https://www.rx-map.com/.

## References

[1] Chen H , Li R, Cleveland A, et al Enhancing data quality in medical concept normalization through large language models. J Biomed Inform. 2025;165:104812. 10.1016/j.jbi.2025.104812 40180205

[2] Peters L , Kapusnik-Uner JE, Bodenreider O. Methods for managing variation in clinical drug names. AMIA Annu Symp Proc. 2010;2010:637-641. 21347056 PMC3041346

[3] Le H , Chen R, Harris S, et al RxNorm for drug name normalization: a case study of prescription opioids in the FDA adverse events reporting system. Front Bioinform. 2023;3:1328613. 10.3389/fbinf.2023.1328613 38250436 PMC10796552

[4] Newman-Griffis D , Divita G, Desmet B, et al Ambiguity in medical concept normalization: an analysis of types and coverage in electronic health record datasets. J Am Med Inform Assoc. 2021;28:516-532. 10.1093/jamia/ocaa269 33319905 PMC7936394

[5] Lagerlund O , Strese S, Fladvad M, et al WHODrug: a global, validated and updated dictionary for medicinal information. Ther Innov Regul Sci. 2020;54:1116-1122. 10.1007/s43441-020-00130-6 32078733 PMC7458889

[6] Ghosh R , Kempf D, Pufko A, et al Automation opportunities in pharmacovigilance: an industry survey. Pharmaceut Med. 2020;34:7-18. 10.1007/s40290-019-00320-0 32036574

[7] Harpaz R , Callahan A, Tamang S, et al Text mining for adverse drug events: the promise, challenges, and state of the art. Drug Saf. 2014;37:777-790. 10.1007/s40264-014-0218-z 25151493 PMC4217510

[8] RxNorm API - APIs. Accessed January 8, 2025. https://lhncbc.nlm.nih.gov/RxNav/APIs/RxNormAPIs.html

[9] ATCDDD - Home. Accessed January 6, 2026. https://atcddd.fhi.no/

[10] Peters L , Kapusnik-Uner JE, Nguyen T, et al An approximate matching method for clinical drug names. AMIA Annu Symp Proc. 2011;2011:1117-1126. 22195172 PMC3243188

[11] Weeks HL , Beck C, McNeer E, et al medExtractR: a targeted, customizable approach to medication extraction from electronic health records. J Am Med Inform Assoc. 2020;27:407-418. 10.1093/jamia/ocz207 31943012 PMC7025369

[12] Luo Y-F , Henry S, Wang Y, et al The 2019 n2c2/UMass lowell shared task on clinical concept normalization. J Am Med Inform Assoc JAMIA. 2020;27:1529-15e1. 10.1093/jamia/ocaa106 32968800 PMC7647359

[13] Hüser M , Doole J, Pinho V, et al A machine learning approach for automating review of a RxNorm medication mapping pipeline output. J Biomed Inform. 2025;170:104909. 10.1016/j.jbi.2025.104909 40945670

[14] Matos J , Gallifant J, Pei J, et al EHRmonize: a framework for medical concept abstraction from electronic health records using large language models. In: Wu S, Shabestari B, Xing L, eds. Applications of Medical Artificial Intelligence. Cham: Springer Nature Switzerland; 2025:210-20.

[15] Sohn S , Clark C, Halgrim SR, et al MedXN: an open source medication extraction and normalization tool for clinical text. J Am Med Inform Assoc. 2014;21:858-865. 10.1136/amiajnl-2013-002190 24637954 PMC4147619

[16] Xu H , Stenner SP, Doan S, et al MedEx: a medication information extraction system for clinical narratives. J Am Med Inform Assoc. 2010;17:19-24. 10.1197/jamia.M3378 20064797 PMC2995636

[17] Savova GK , Masanz JJ, Ogren PV, et al Mayo clinical text analysis and knowledge extraction system (cTAKES): architecture, component evaluation and applications. J Am Med Inform Assoc. 2010;17:507-513. 10.1136/jamia.2009.001560 20819853 PMC2995668

[18] Meldau E-L , Bista S, Rofors E, et al Automated drug coding using artificial intelligence: an evaluation of WHODrug koda on adverse event reports. Drug Saf. 2022;45:549-561. 10.1007/s40264-022-01162-7 35579817 PMC9114093

[19] RxClass API - APIs. Accessed January 9, 2026. https://lhncbc.nlm.nih.gov/RxNav/APIs/RxClassAPIs.html

[20] Ruggles S , Flood S, Sobek M, et al IPUMS USA: Version 16.0. 2025.

[21] Heaton RK , Clifford DB, Franklin DR, et al HIV-associated neurocognitive disorders persist in the era of potent antiretroviral therapy: CHARTER study. Neurology. 2010;75:2087-2096. 10.1212/WNL.0b013e318200d727 21135382 PMC2995535

[22] Welcome to the HNRP. Accessed January 8, 2025. https://hnrp.hivresearch.ucsd.edu/

[23] Morgello S , Gelman BB, Kozlowski PB, et al The national NeuroAIDS tissue consortium: a new paradigm in brain banking with an emphasis on infectious disease. Neuropathol Appl Neurobiol. 2001;27:326-335. 10.1046/j.0305-1846.2001.00334.x 11532163

[24] Chalabianloo N , Abdullah SS, Omrani MA, et al Enhancing adverse drug reaction data quality in Canada: a high-precision pipeline for medication name standardization and enrichment. Plos One,. 2025; 20: e0331940. 10.1371/journal.pone.0331940 40997135 PMC12463261

