# Supplementary material for: RxMap: an LLM-assisted tool for medication normalization
Source: JAMIA Open. 2026 May 30;9(3):ooag085. doi: 10.1093/jamiaopen/ooag085 (PMC13221971; doi:10.1093/jamiaopen/ooag085)
Supplement: ooag085_Supplementary_Data [file ooag085_supplementary_data.zip › 23-May-2026_063805_RxMap_SI.docx]

**Supplementary Information of “RxMap: An LLM-Assisted Tool for Medication Normalization”**

Table of Contents

[S1 IMPLEMENTATION DETAILS 2](#_Toc225777824)

[S1.1 System Architecture 2](#_Toc225777825)

[S1.2 External Dependencies and Versioning 3](#_Toc225777826)

[S1.3 Caching Strategy 3](#_Toc225777827)

[S2 HYBRID NORMALIZATION 4](#_Toc225777828)

[S2.1 Phase 1: Dual Candidate Generation 4](#_Toc225777829)

[S2.1.1 Deterministic Candidate Generation 4](#_Toc225777830)

[S2.1.2 LLM-Assisted Candidate Generation 5](#_Toc225777831)

[S2.2 Phase 2: Hierarchical Candidate Selection 6](#_Toc225777832)

[S2.2.1 Empirical Verification of Confidence Categories 10](#_Toc225777835)

[S2.2.2 Ablation Analysis of Weak Prior Integration in LLM 10](#_Toc225777836)

[S3 POST-NORMALIZATION ATC CLASSIFICATION 11](#_Toc225777838)

[S4 USER INTERFACE AND BATCH REVIEW WORKFLOWS 15](#_Toc225777839)

[S4.1 Upload File for Batch Normalization 18](#_Toc225777840)

[S4.2 Automatic Mappings Preview and Download 18](#_Toc225777841)

[S4.3 Manual Drug Mapping Review 19](#_Toc225777842)

[S4.4 Manual ATC Classification Review 19](#_Toc225777843)

[S4.5 Reviewed Mappings Summary and Download 20](#_Toc225777844)

[S4.6 Effect of Manual Review on Performance 20](#_Toc225777845)

[S5 OUTPUT FORMATS, PROVENANCE, AND REPRODUCIBILITY 20](#_Toc225777846)

[S5.1 Output files and schemas 21](#_Toc225777847)

[S5.1.1 Automatic and restore-session mapping outputs 21](#_Toc225777848)

[S5.1.2 Finalized (reviewed) drug-level mapping outputs 22](#_Toc225777849)

[S5.2 Provenance and auditability 23](#_Toc225777850)

[S5.3 Reproducibility considerations 23](#_Toc225777851)

[S6 DATA AND GOLD-STANDARD MAPPINGS 23](#_Toc225777852)

# IMPLEMENTATION DETAILS

## System Architecture

RxMap is implemented as a web-based system that supports both single-string queries and batch processing of medication lists provided in CSV or XLSX formats. The system produces structured, normalized outputs suitable for downstream pharmacoepidemiologic and drug utilization analyses.

At a high level, data flow through three processing stages: (i) input preprocessing, (ii) ingredient-level normalization, and (iii) post-normalization ATC annotation. Preprocessing applies minimal string sanitization to facilitate consistent querying, deduplication, and caching. Preprocessed strings are then passed to the normalization stage, which comprises two phases: (1) dual candidate generation, in which candidate ingredient sets are obtained independently from deterministic RxNorm matching and constrained LLM-assisted parsing; and (2) hierarchical candidate selection, in which agreement checks and subset resolution logic are used to reconcile candidates into finalized RxNorm ingredient- or multi-ingredient-level concepts (RxCUIs). Following normalization, ATC classification is applied as a post-hoc annotation step to mapped RxNorm concepts.

## External Dependencies and Versioning

The analyses reported in this study used RxNorm terminology released on 12/01/2025, incorporating ATC mappings based on the ATC release dated 02/10/2025. The LLM-assisted components were instantiated using Google’s *gemini-2.0-flash* and OpenAI’s *gpt-5-nano* models. In deployed use, RxMap queries the live RxNorm API, allowing users to access current terminology releases without requiring local installation or version management. To support reproducibility in research settings, all normalization results are associated with metadata specifying the RxNorm release version, LLM provider, and model identifier used at the time of mapping.

## Caching Strategy

To support efficient reprocessing and reproducible normalization, RxMap implements a cache at the medication-entry level. Cache keys are derived from preprocessed medication strings, enabling stable reuse of results across repeated inputs. All automatically generated mappings, including high-confidence exact matches, normalized matches, and subset-based matches, are stored in the cache.

For each cached entry, RxMap records the LLM provider and model identifier used during candidate generation, ensuring that repeated runs on identical inputs yield consistent outputs even when multiple LLM backends are supported. Post-normalization ATC annotations are cached alongside RxNorm mappings, enabling deterministic reuse of both normalization and classification results across sessions. This caching strategy provides computational efficiency while supporting auditability and reproducibility in batch normalization workflows.

# HYBRID NORMALIZATION

The hybrid normalization module maps each preprocessed medication string to standardized RxNorm ingredient-level (IN) or multi-ingredient (MIN) concepts, with RxNorm serving as the authoritative reference vocabulary. To improve robustness and coverage while preserving clinical validity, normalization is organized into two phases: **dual candidate generation** and **hierarchical candidate selection** (Supplementary Figure 1).

## Phase 1: Dual Candidate Generation

Phase 1 produces two independent candidate ingredient representations for each medication string: a deterministic RxNorm-derived candidate and an LLM-assisted candidate. These complementary candidates are reconciled in Phase 2.

### *Deterministic Candidate Generation*

Preprocessed medication strings are submitted to the RxNorm API using the getApproximateMatch function. Queries are configured to return only the top-ranked candidate, relying on RxNorm’s internal scoring to prioritize the most plausible concept.

If the returned concept corresponds directly to an IN or MIN concept, it is passed forward to the selection stage. Otherwise, the concept is normalized to its generic ingredient representation. For active non-generic concepts, normalization is performed by resolving related ingredient or multi-ingredient concepts using the getAllRelatedInfo or getRelatedByRelationship endpoints. For obsolete concepts, the getRxcuiHistoryStatus endpoint is queried to identify successor ingredient concepts. The names of derived ingredients are concatenated into a composite query string and recursively re-submitted to getApproximateMatch to obtain a current IN or MIN concept. This procedure ensures that all deterministic candidates entering the selection stage are active, generic RxNorm ingredient representations.


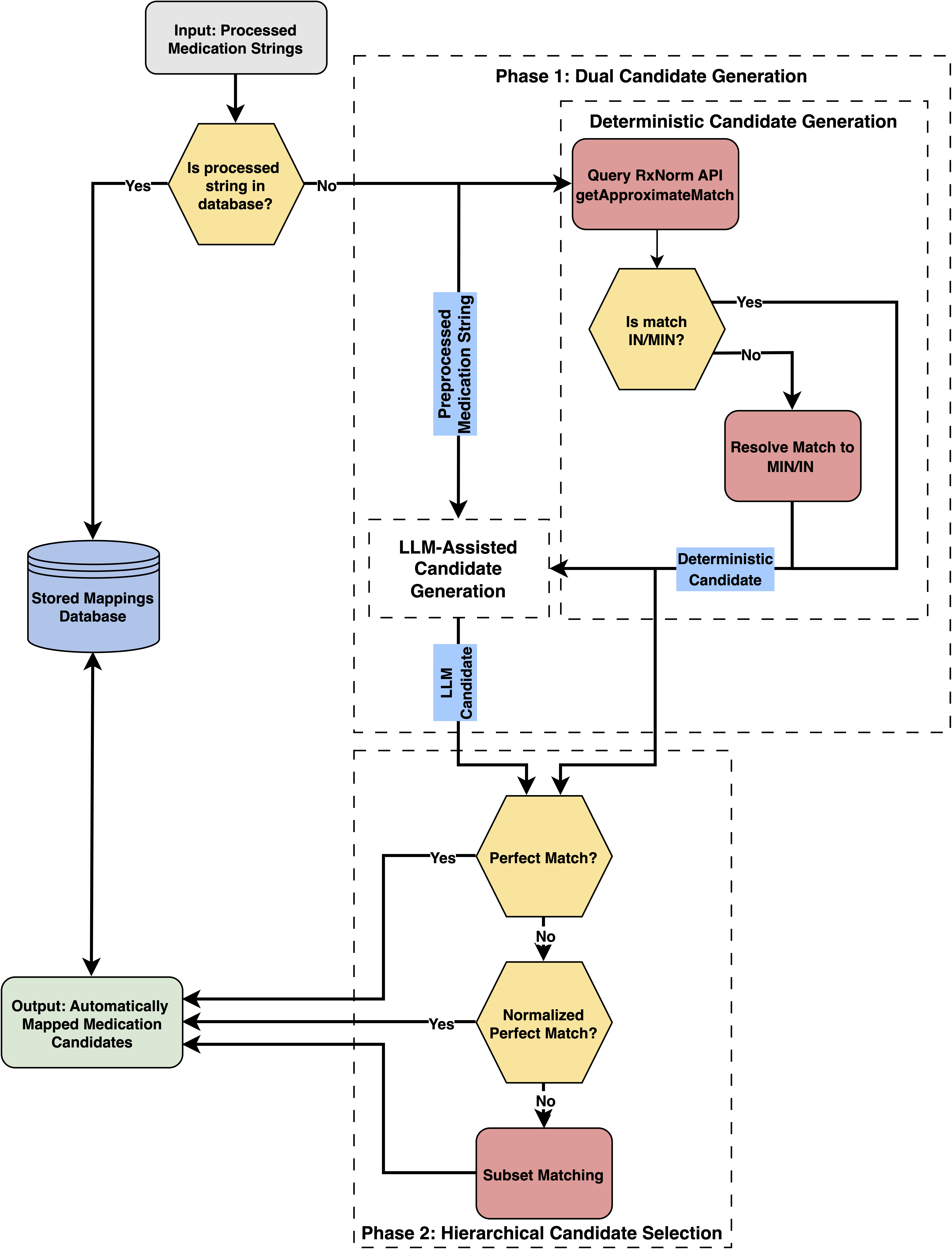


**Supplemental Figure 1.** Hybrid Normalization architecture.

### *LLM-Assisted Candidate Generation*

The LLM is used as a secondary inference layer to standardize free-text medication strings into generic RxNorm ingredient names. The model is provided with the preprocessed medication string and, when available, the deterministic RxNorm candidate. The deterministic candidate serves only as a weak prior and is included to constrain generation when consistent with the raw input. The model is explicitly instructed to treat the raw medication string as the primary source of truth and to disregard the deterministic candidate when disagreement or ambiguity is present.

The LLM is prompted using a fixed few-shot instruction template with representative examples. The prompt directs the model to expand brand or proprietary names to their generic active ingredients and to represent combination products using forward-slash delimiters consistent with RxNorm conventions. Inference is performed using deterministic decoding. The output is restricted to a single-line, structured string containing only standardized ingredient names; no free text or explanatory content is permitted. Prompt templates and examples are provided in Supplementary Figure 2.

## Phase 2: Hierarchical Candidate Selection

After deterministic and LLM-assisted candidate generation, the backend applies a multi-stage mapping procedure to maximize agreement with the original medication entry while maintaining broad match coverage. Two ingredient sets are constructed: a deterministic set derived from the RxNorm and an LLM-derived set obtained by parsing the LLM output, with ingredient sets defined by splitting string representations exclusively on forward slashes. These sets form the basis for all downstream matching logic. The backend first evaluates a perfect match (high confidence match). A perfect match is defined as exact equivalence between the deterministic ingredient set and the LLM-derived ingredient set. Equivalence requires identical ingredient membership.

If a perfect match is not identified, the system evaluates a normalized perfect match (moderate confidence match). In this case, both ingredient sets are first normalized using canonical RxNorm ingredient representations after which exact equivalence is reassessed. Here,


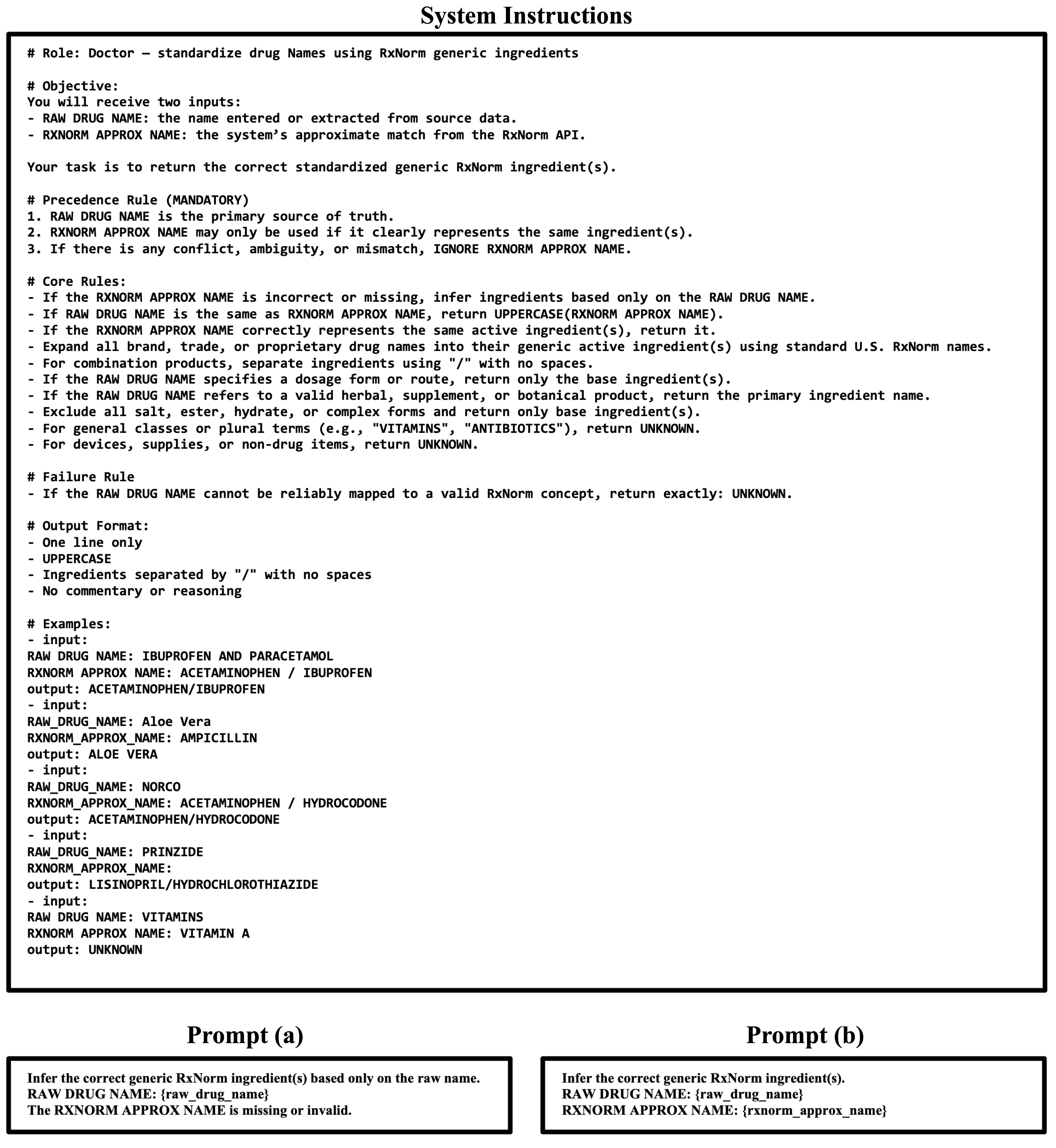


**Supplemental Figure 2.** System instructions and prompt templates used to guide the large language model (LLM) in standardizing raw drug names into RxNorm generic ingredient representations. Prompt (a) is used when no deterministic candidate is available and (b) when a when a deterministic candidate is available.

both ingredient sets are normalized at the level of individual ingredient strings via the deterministic mapping procedure described in S2.1.1 to their canonical RxNorm ingredient names (e.g., “VITAMIN K1” → “PHYTONADIONE”; “VITAMIN K2” → “MENAQUINONE”; “ACETYLSALICYLIC ACID” → “ASPIRIN”; “
CODEINE PHOSPHATE” → “CODEINE”). If the normalized sets are identical, the match is finalized. A normalized perfect match therefore captures cases in which the same underlying ingredients are expressed using different lexical forms but correspond to the same set.

When neither a perfect match nor a normalized perfect match is identified, the backend performs subset matching (low-confidence match). In this mode, the normalized LLM-derived ingredient set is decomposed into progressively smaller ingredient subsets, formed and evaluated in descending size order. Each subset is evaluated deterministically against RxNorm concepts. Subset matching is applied iteratively, selecting valid single-ingredient or multi-ingredient combinations and removing the matched ingredients from the remaining set until no ingredients remain or no further valid combinations can be resolved. By prioritizing the largest possible subsets before considering smaller ones, this approach ensures that combination products are decomposed in a structure-preserving manner rather than prematurely collapsing into unrelated single-ingredient mappings, thereby maintaining alignment with the original raw medication. representation. Algorithm 1 shows pseudocode for the subset matching logic.


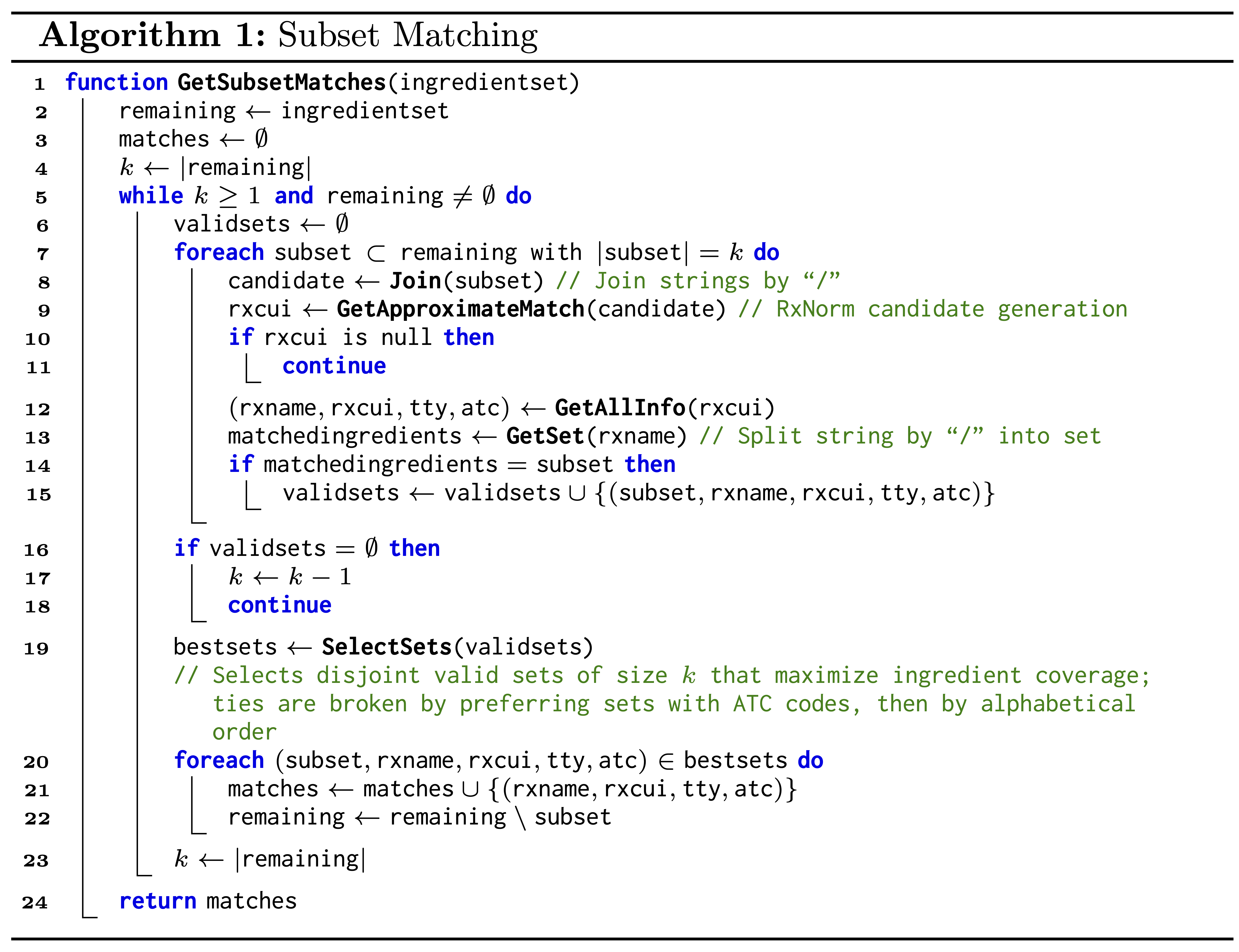


To better understand how the function works, consider the following example:

**Example:** Suppose the raw medication string is "ASA/ACETAMINOPHEN/CAFFEINE/COD". The deterministic mapping returns the set {ACETAMINOPHEN, ASPIRIN, CAFFEINE}, which is incomplete. The LLM, however, correctly generates the candidate set {ACETAMINOPHEN, ASPIRIN, CAFFEINE, CODEINE}. Because the sets are not equivalent, there is no perfect match. Further, there cannot be a normalized perfect match since the number of ingredients differs. Thus, RxMap applies subset matching on the LLM-generated ingredient set.

The subset matching algorithm begins by attempting to match all four ingredients as a single drug concept (ACETAMINOPHEN/ASPIRIN/CAFFEINE/CODEINE). Although a five-ingredient RxNorm concept exists that covers all four ingredients (ACETAMINOPHEN/ASPIRIN/CAFFEINE/CODEINE/SALICYLAMIDE), it includes an additional ingredient (SALICYLAMIDE) and therefore fails the exact-ingredient requirement.

The algorithm then reduces the subset size to three and evaluates all three-ingredient combinations. At this level, several valid exact matches exist, including {ASPIRIN, CAFFEINE, CODEINE}, {ACETAMINOPHEN, CAFFEINE, CODEINE}, {ACETAMINOPHEN, ASPIRIN, CODEINE}, and {ACETAMINOPHEN, ASPIRIN, CAFFEINE}. Because multiple subsets cover the same number of ingredients, the algorithm applies tie-breaking rules: all candidates provide equal coverage, but {ACETAMINOPHEN, ASPIRIN, CAFFEINE} is preferred because it has associated ATC codes and is alphabetically earlier than competing triples.

This three-ingredient match is selected and its ingredients are removed from the remaining set. The algorithm then continues with the leftover ingredient {CODEINE}, which is successfully matched as a single-ingredient concept. The final result is two RxNorm concepts: the three-ingredient combination ACETAMINOPHEN/ASPIRIN/CAFFEINE plus the single-ingredient CODEINE. This decomposition preserves the structure of the original medication while ensuring all mappings correspond to valid RxNorm concepts.

### *Empirical Verification of Confidence Categories*

To validate that match type provides a meaningful proxy for mapping confidence, RxMap’s performance stratified by match type (perfect, normalized perfect, and subset) was evaluated across two datasets: IPUMS MEPS and an independent harmonized dataset CHARTER/NNTC/HNRP. Performance was assessed at both the RxCUI and ingredient levels using precision, recall, and F1-score, as seen in Supplementary Table 1.

Across both datasets, match type consistently aligns with empirical performance: perfect matches (high confidence) achieve near-ceiling precision and recall, normalized perfect matches (moderate confidence) maintain high accuracy with slight degradation, and subset matches (low confidence) exhibit lower but still substantial performance. These results support the use of match type as a structured, rule-based confidence categorization for prioritizing manual review.

**Supplementary Table 1.** Performance of RxMap (Gemini-2.0-Flash) stratified by confidence level (match type) across IPUMS MEPS and CHARTER/NNTC/HNRP datasets at the RxCUI and ingredient levels.

|  |  | RxCUI | | | Ingredient | | |
| --- | --- | --- | --- | --- | --- | --- | --- |
| Dataset | Performance Metrics | Low  (Subset) | Moderate (Normalized Perfect) | High  (Perfect) | Low  (Subset) | Moderate (Normalized Perfect) | High  (Perfect) |
| IPUMS MEPS | F1 | 0.853 | 0.995 | 0.997 | 0.907 | 0.996 | 0.998 |
|  | Precision | 0.835 | 0.996 | 0.998 | 0.899 | 0.997 | 0.998 |
|  | Recall | 0.872 | 0.993 | 0.997 | 0.916 | 0.995 | 0.998 |
| CHARTER/NNTC/  HNRP | F1 | 0.789 | 0.960 | 0.998 | 0.830 | 0.964 | 0.997 |
|  | Precision | 0.759 | 0.931 | 0.998 | 0.821 | 0.938 | 0.998 |
|  | Recall | 0.822 | 0.991 | 0.998 | 0.839 | 0.992 | 0.996 |

### *Ablation Analysis of Weak Prior Integration in LLM*

We conduct an ablation study to assess the contribution of the deterministic candidate as a weak prior. In the full RxMap pipeline, the LLM operates with two prompting modes as seen in Supplemental Figure 2. Prompt (a) generates candidates directly from the raw drug name, and Prompt (b) incorporates a deterministic candidate as auxiliary guidance when a match is available. For the ablation, we remove the deterministic candidate as a weak prior by disabling Prompt (b) and running the LLM exclusively with Prompt (a) for all inputs. This setup isolates the effect of the deterministic candidate by comparing performance with and without its inclusion, using the best-performing configuration (gemini-2.0-flash) on the CHARTER/NNTC/HNRP dataset, as summarized in Supplementary Table 2.

The results indicate that incorporating the deterministic candidate as a weak prior provides a modest but consistent improvement, particularly in recall, while preserving the LLM’s ability to correct erroneous matches. This suggests that the deterministic candidate provides useful contextual guidance that helps the LLM recover correct mappings more reliably.

**Supplementary Table 2**. Ablation study results comparing RxMap performance with and without deterministic candidate as weak prior, evaluated on the CHARTER/NNTC/HNRP dataset using the gemini-2.0-flash model.

|  |  | RxCUI | | Ingredient | |
| --- | --- | --- | --- | --- | --- |
| Dataset | Performance Metrics | RxMap w/o prior (gemini-2.0-flash) | RxMap  (gemini-2.0-flash) | RxMap w/o prior (gemini-2.0-flash) | RxMap  (gemini-2.0-flash) |
| CHARTER/NNTC/  HNRP | F1 | 0.946 | 0.956 | 0.950 | 0.953 |
|  | Precision | 0.955 | 0.962 | 0.965 | 0.966 |
|  | Recall | 0.937 | 0.950 | 0.936 | 0.941 |

# POST-NORMALIZATION ATC CLASSIFICATION

Anatomical Therapeutic Chemical (ATC) annotations are assigned to normalized RxNorm concepts using a hierarchical, validation-driven procedure that integrates information from the RxNorm and RxClass APIs. ATC assignment is performed strictly as a post-normalization step and does not alter the underlying RxNorm identity resolution.

For each automatically mapped RxNorm concept, the system first queries the RxNorm API (getAllProperties) to retrieve all available concept properties associated with the RxCUI. The returned properties include names, values, and categories. The CODES property category is examined for the presence of ATC code attributes. When ATC codes are directly available within RxNorm concept properties, these codes are assigned preferentially, as they represent authoritative, concept-level annotations curated within RxNorm.

When ATC codes are not present in RxNorm properties, ATC membership is inferred using the RxClass API. Specifically, the getClassByRxNormDrugId endpoint is queried using the ATC and ATCprod relationship sources to retrieve candidate ATC classes associated with the RxNorm concept. Because RxClass relationships may link related but non-identical drug concepts, all inferred ATC assignments are subjected to an additional validation step prior to acceptance.

This validation enforces ingredient-level equivalence between the normalized RxNorm concept and the RxNorm concept associated with each candidate ATC assignment. Active ingredient sets are derived by querying the RxNorm API (getRelatedByType with term type IN) for both the normalized concept and the RxClass-linked concept. An ATC code is assigned only if the two ingredient sets are identical. This strict equivalence requirement ensures pharmacologic consistency and prevents the propagation of ATC codes across concepts that differ in ingredient composition, dose form, or formulation.

Through this two-stage strategy of prioritizing direct ATC annotations from RxNorm when available and otherwise applying conservative, ingredient-validated inference from RxClass, the system balances classification coverage with correctness while maintaining reproducible ATC assignment behavior. A schematic overview of the ATC classification workflow is provided in Supplementary Figure 3.

In addition to full ATC assignments derived through direct annotation or strict ingredient-level validation, the system also accounts for scenarios in which complete classification is not attainable for a normalized concept. Partial coverage arises in cases where an automatically normalized MIN concept does not have a directly assigned ATC code and does not satisfy strict ingredient-level equivalence criteria for any single ATC class. In such situations, the system performs a structured subset evaluation of the ingredient set. Specifically, all non-empty proper subsets of the active ingredient set are generated and individually evaluated for ATC eligibility using the same validation-driven procedure described above. For each subset, ingredient-level equivalence is enforced against candidate ATC-linked RxNorm concepts to ensure pharmacologic consistency. Subsets that successfully meet equivalence criteria are assigned their corresponding ATC codes as potential annotations for the original combination product. These annotations are explicitly designated as partial coverage to distinguish them from full ingredient-matched assignments. Partial coverage therefore indicates that while no ATC code exists for the exact multi-ingredient mapped candidate, valid ATC classifications are available for one or more constituent components.


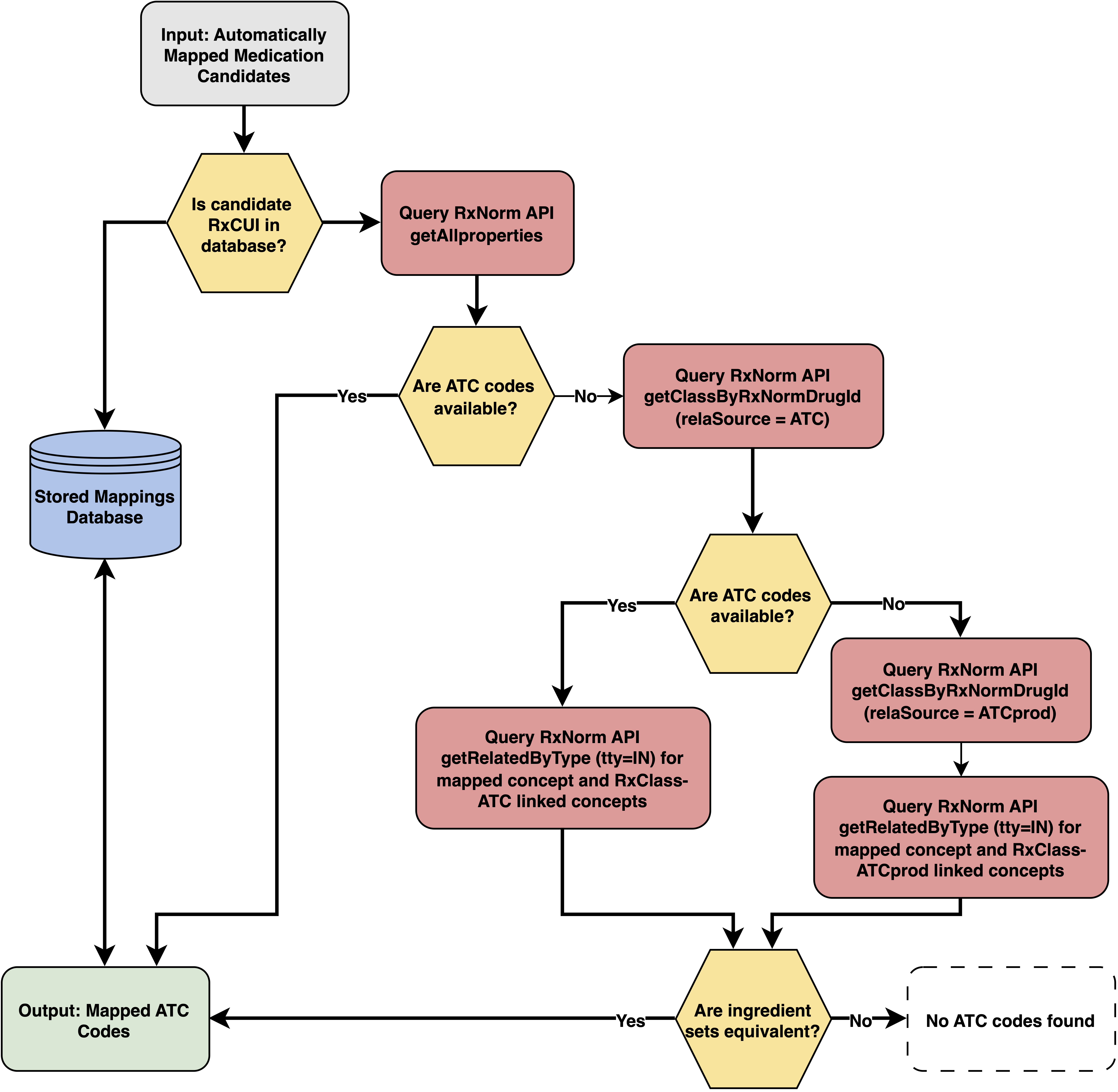


**Supplemental Figure 3.** ATC Assignment architecture.

For example, the MIN product CALAMINE / DIMETHICONE / ZINC OXIDE illustrates this behavior. Although this product is a valid RxNorm MIN concept, no ATC code exists for the exact three-ingredient formulation. Consequently, the system evaluates all proper subsets. Two-ingredient subsets such as CALAMINE / ZINC OXIDE and ZINC OXIDE **/** DIMETHICONE are identified as RxNorm concepts with valid ATC classifications. Individual ingredients also yield valid ATC assignments (e.g., DIMETHICONE and ZINC OXIDE). Each of these subset concepts passes ingredient-level equivalence validation and is therefore retained as a partial coverage annotation.

# USER INTERFACE AND BATCH REVIEW WORKFLOWS

RxMap provides a browser‑based front-end interface designed for high‑throughput curation of medication mappings while preserving fine‑grained control over individual decisions. The batch review workflow is organized as a linear pipeline: users upload a CSV or XSLX file, run automated mapping, and can immediately download the automatic MIN/IN drug mappings and expanded IN-only mappings with ATC annotations as an XSLX file. If manual review of output desired, users proceed to review drug‑level candidates, review ATC‑level candidates, and then export the finalized results. Supplementary Figures 4-9 shows screenshots for of the pipeline at each step.


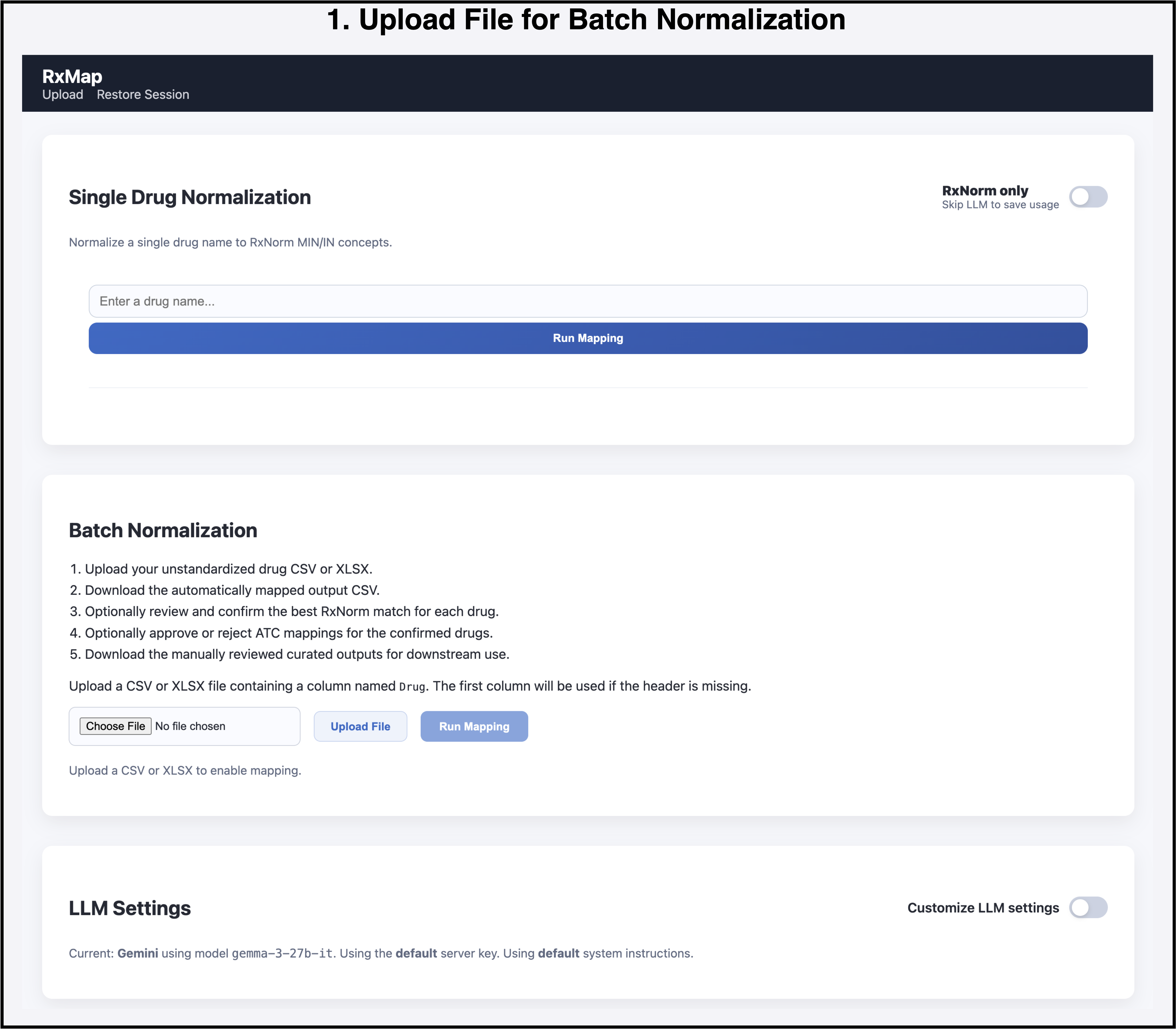


**Supplemental Figure 4.** RxMap front-end user interface upload page.


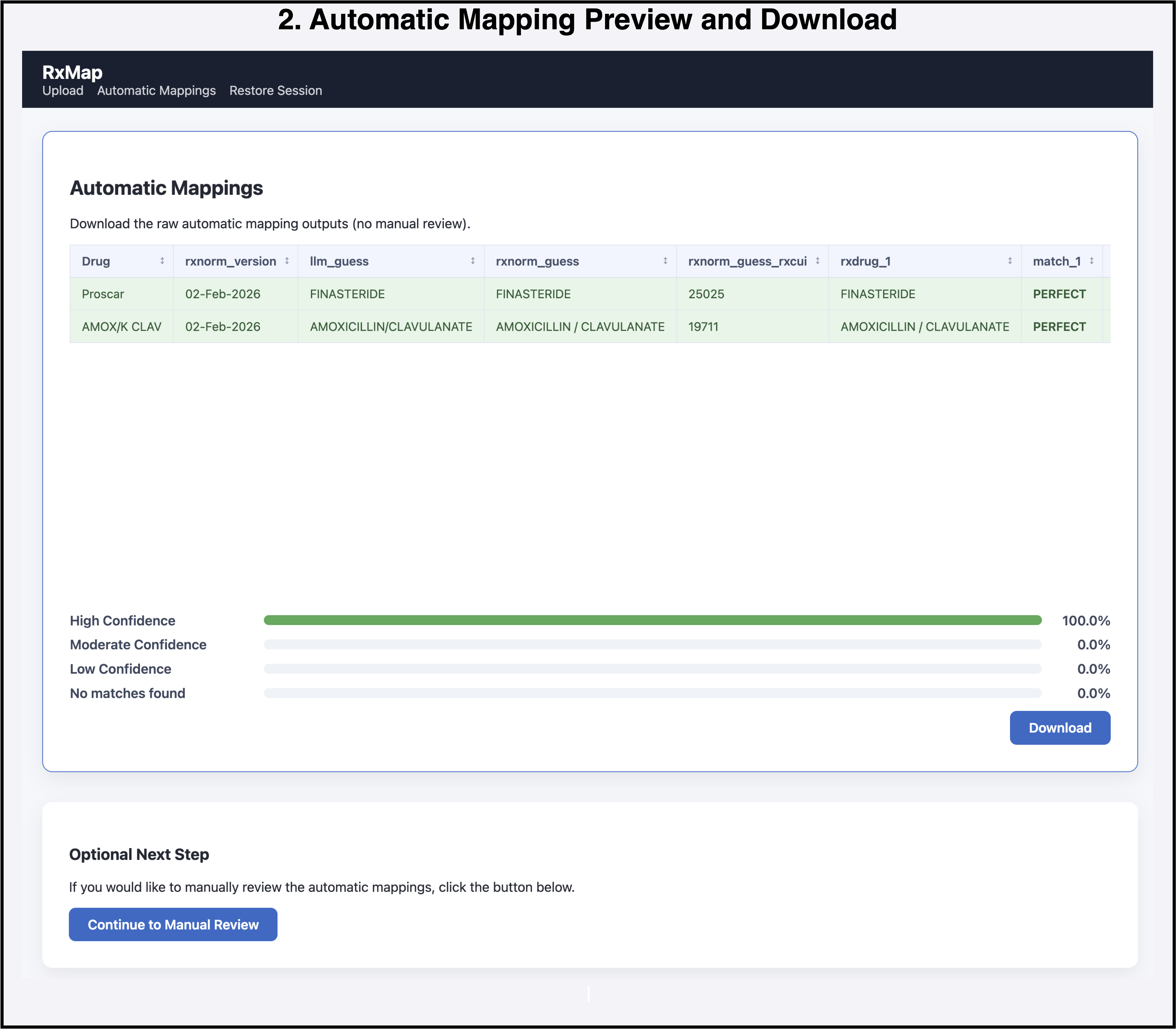


**Supplemental Figure 5.** RxMap front-end user interface automatic mappings output page.


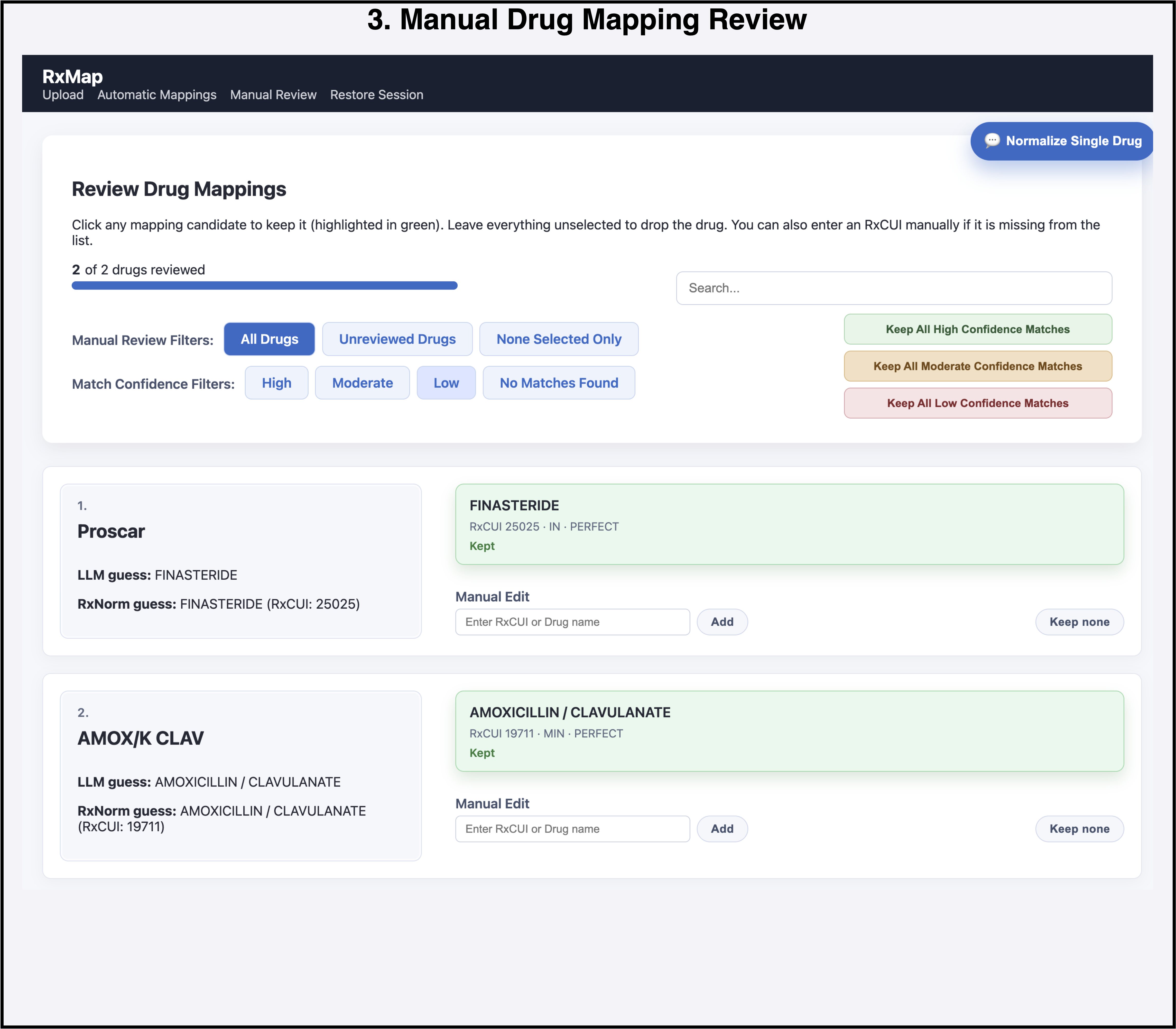


**Supplemental Figure 6.** RxMap front-end user interface automatic mappings manual review page.


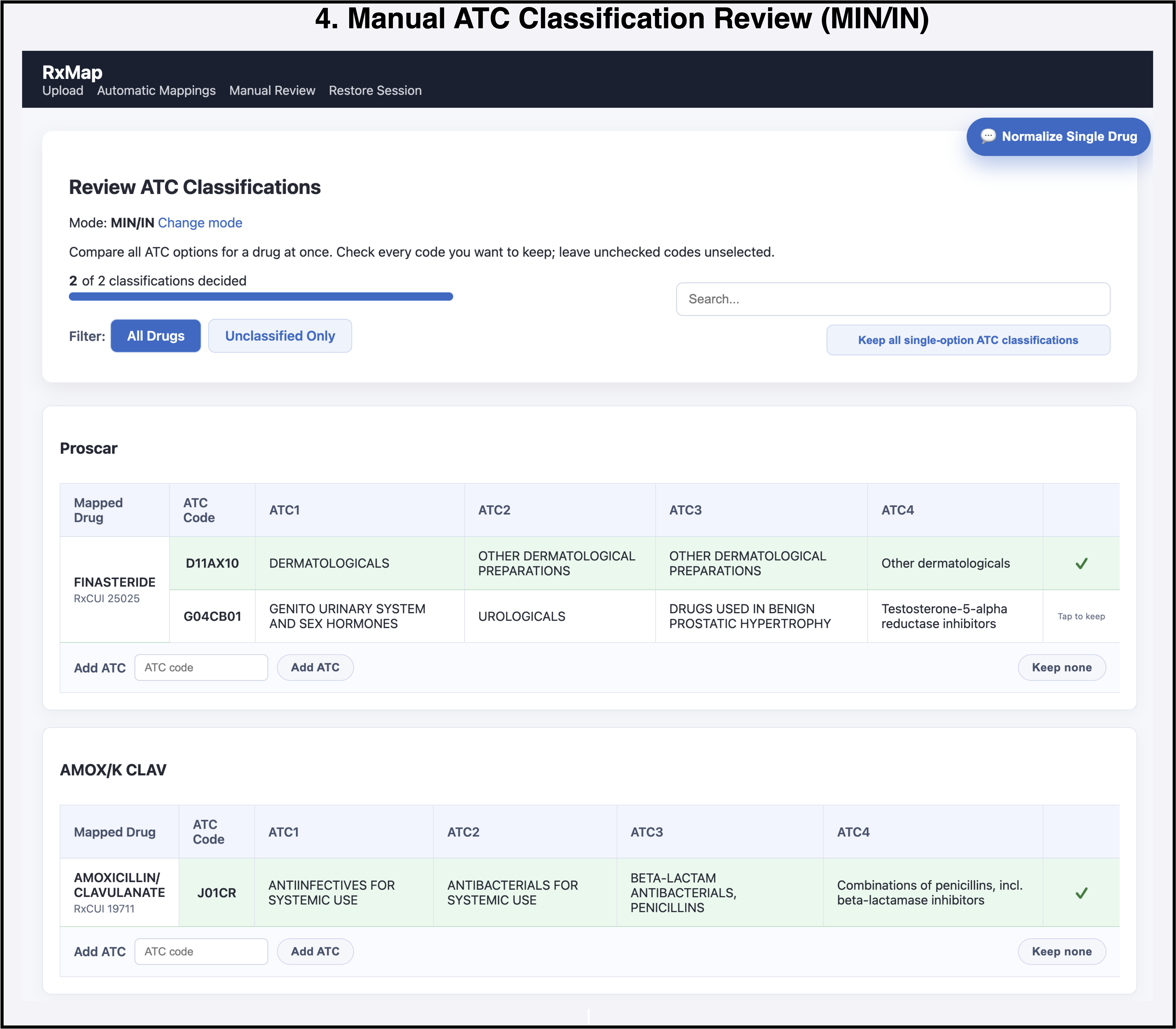


**Supplemental Figure 7.** RxMap front-end user interface ATC annotation manual review page for MIN/IN mappings.


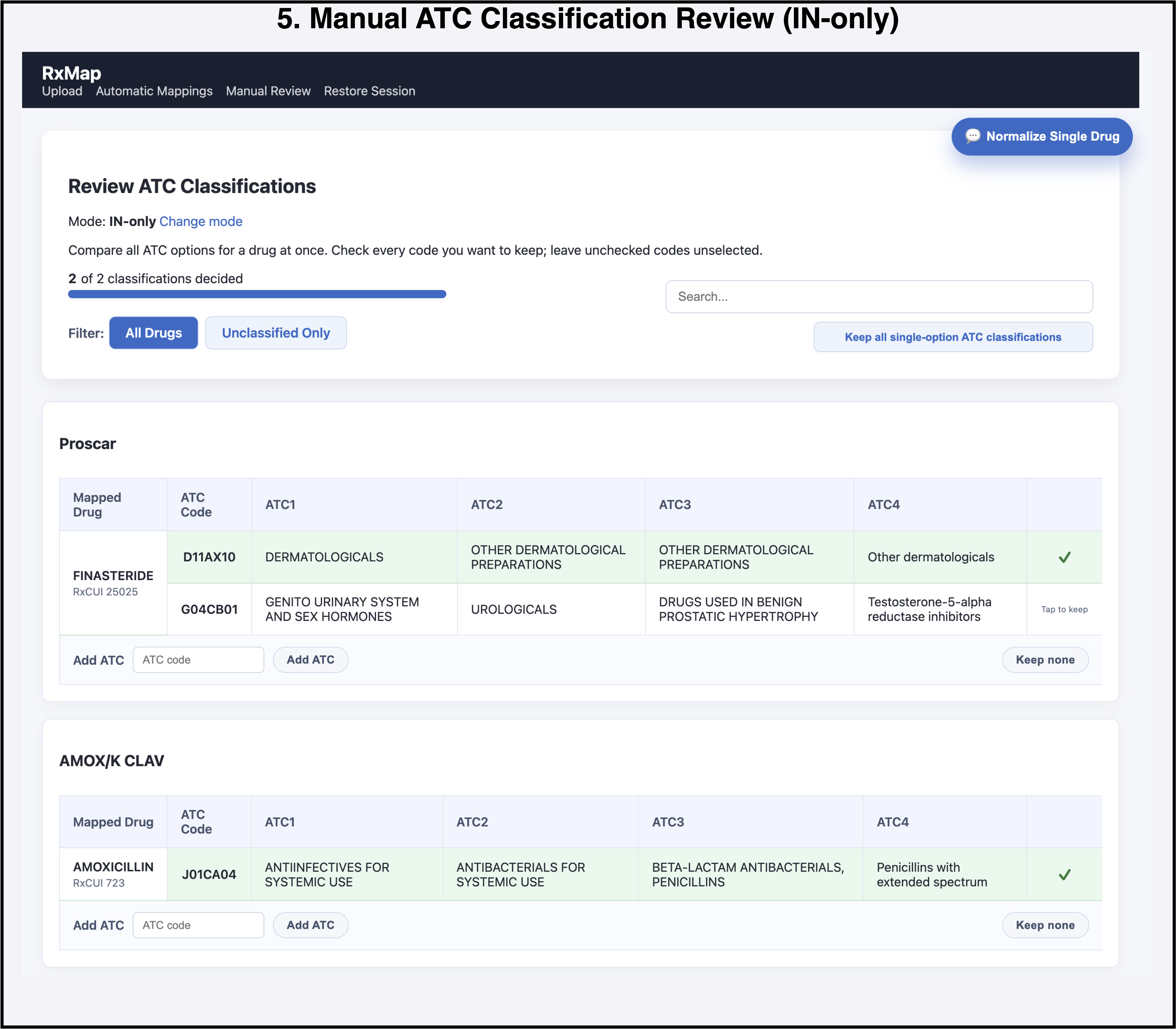


**Supplemental Figure 8.** RxMap front-end user interface ATC annotation manual review page for IN-only mappings.


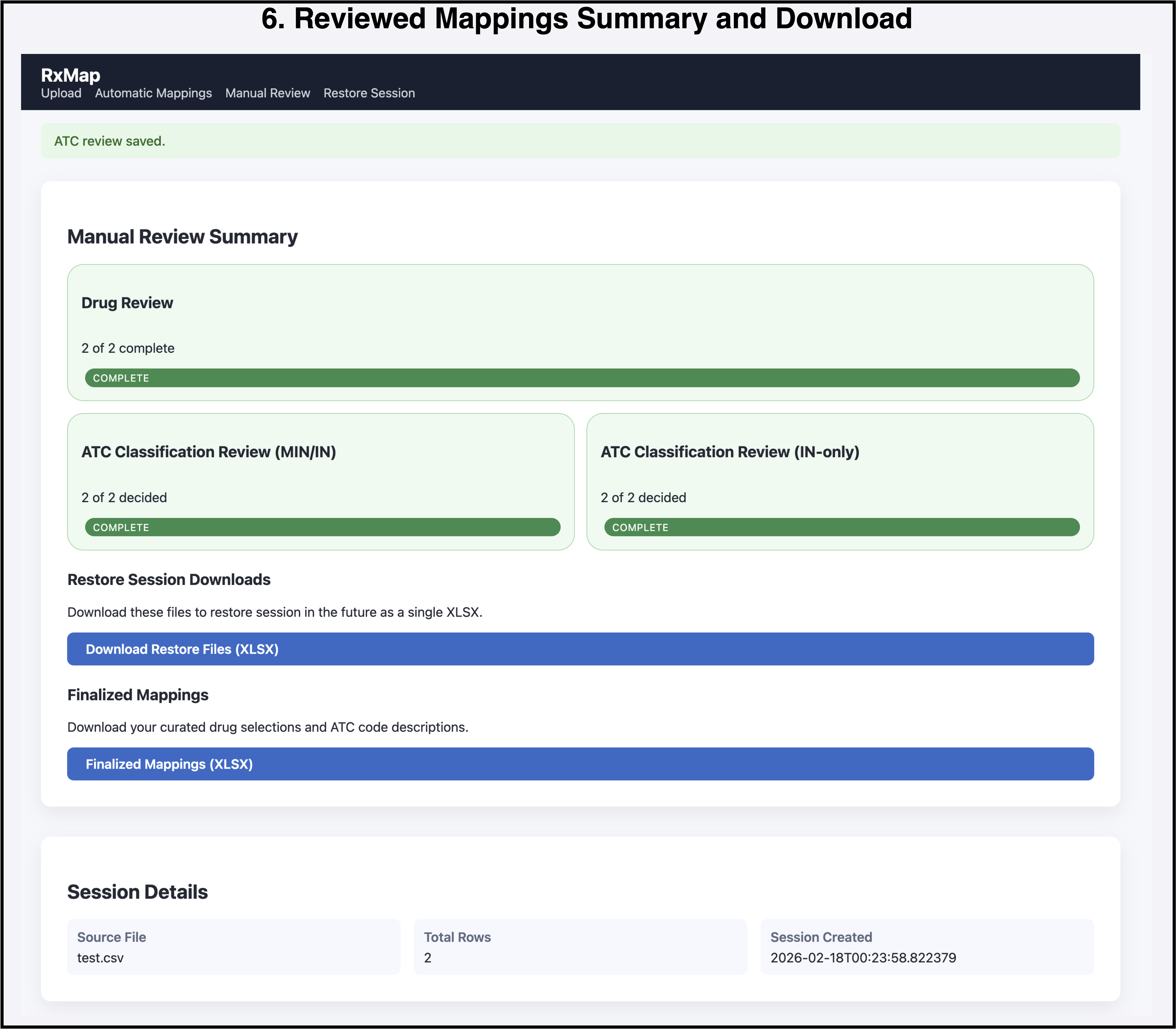


**Supplemental Figure 9.** RxMap front-end user interface summary page with restore session and finalized mappings downloads.

## Upload File for Batch Normalization

The upload and configuration view serves as the entry point for batch processing (Supplementary Figure 4). Users submit a CSV/XLSX file containing drug names in a column named Drug. On clicking run mapping, the system performs automated mapping and ATC classification annotation, returning a XLSX of the results. Before running the mapping, users can either run RxMap freely with the default system settings (Gemini’s gemma-3-27b-it model), or optionally provide an API key for accessing additional LLM providers and models.

## Automatic Mappings Preview and Download

After the automatic mapping has been completed, users are brought to the Automatic Mappings page of the web application (Supplementary Figure 5). On this page, users can preview the output with a breakdown of the percentage of high confidence, moderate confidence, and low confidence mappings. Occasionally, the system will not find any candidate mappings for the input drug string, which the percentage breakdown also includes. The downloadable Excel datasheet contains both MIN/IN mappings and an IN‑only view in which MIN drug mappings are expanded to their ingredient representations, providing users with ingredient‑level mappings alongside the original mapping results.

## Manual Drug Mapping Review

If users wish to manually review the automatic mappings, the drug mapping review page (Supplementary Figure 6) provides this option. Users see the raw input drug string alongside RxNorm and LLM candidate guesses from the dual candidate generation step, but only the candidates selected by the hierarchical candidate selection phase are shown for verification. Each candidate is listed with its name, RxCUI, term type, and match type (perfect, normalized perfect, or subset). Users may keep one or multiple candidates, explicitly keep none, or manually edit candidates when automated mapping is incomplete or incorrect. The interface continuously displays progress (reviewed vs. total) and supports search and filtering, enabling focus on subsets such as unreviewed entries or mapping confidence tiers. Bulk actions (e.g., keep‑all for a confidence tier) accelerate large‑scale curation while still allowing per‑drug overrides.

## Manual ATC Classification Review

The automatic post-hoc ATC annotations can also be manually reviewed in the web application. After drug mapping review, only accepted candidates with available ATC codes are shown, and each candidate’s ATC options are listed in a table that includes the code and its hierarchical context (ATC1–ATC4), allowing rapid comparison of clinical categories. Users can keep automatically annotated ATC codes, edit these ATC codes, or keep none when no codes apply; an auto‑keep action can accept single‑option groups in bulk. The ATC review is available in two modes. MIN/IN review evaluates ATC annotations associated with the accepted MIN/IN drug mappings and is used when multi-ingredient products or specific RxNorm matches should determine ATC selection (Supplemental Figure 7). IN-only ATC review (Supplemental Figure 8) limits decision-making to expanded ingredient-level mappings, which is useful when users want to analyze combination products in their simplified, expanded-ingredient form.

## Reviewed Mappings Summary and Download

After review, the Summary page (Supplemental Figure 9) consolidates progress across drug and ATC stages and provides the outputs needed for downstream use. It offers a restore‑session export that lets users save progress and later resume by re‑uploading the prior export files, enabling extended, multi‑session curation without losing decisions. It also provides the finalized mappings download, which contains the curated drug selections and ATC annotations ready for analysis, reporting, or integration into other pipelines.

## Effect of Manual Review on Performance

Since RxMap has an optional review interface, we evaluated its effectiveness in resolving low-confidence cases. In this evaluation, 949 of 22,624 entries in the IPUMS MEPS dataset were flagged for review under conservative confidence thresholds. Incorporating user confirmation for these cases yields near-complete coverage (F1 > 0.995), illustrating the practical utility of the review interface for quality control in large-scale normalization workflows while retaining a fully automated default operating mode for most inputs.

# OUTPUT FORMATS, PROVENANCE, AND REPRODUCIBILITY

RxMap produces three primary categories of outputs corresponding to distinct stages of the workflow: automatic mapping exports generated immediately after mapping but prior to manual review, restore-session exports generated during manual review to preserve candidate selection and editing state, and finalized mapping exports generated post manual review, representing the authoritative curated state. Automatic and restore-session outputs share closely related schemas reflecting candidate-level provenance, whereas finalized outputs retain only validated mappings and omit workflow-specific metadata.

## Output files and schemas

### *Automatic and restore-session mapping outputs*

Automatic mapping and restore-session drug-level sheets constitute the core computational output of RxMap. Each row corresponds to a single free-text drug string mapped to one or more candidate RxNorm concepts. Candidate-specific columns follow an indexed convention, where *i* denotes a distinct candidate mapping associated with the input drug string. Restore-session outputs preserve the same structural logic while reflecting the most recent machine-derived state used to reconstruct a session. Supplemental Table 3 explicitly shows the schema for both exports.

**Supplementary Table 3.** Drug-level mapping output schema for automatic and restore-session exports

| **Column** | **Description** |
| --- | --- |
| *Drug* | Original free-text medication string. |
| *llm_guess* | LLM-assisted generated candidate string. |
| *rxnorm_guess* | RxNorm deterministically generated candidate string. |
| *rxnorm_guess_rxcui* | RxNorm Concept Unique Identifier (RxCUI) for *rxnorm_guess*. |
| *rxdrug_i* | RxNorm drug name for candidate *i* chosen via hierarchical candidate selection. |
| *rxcui_i* | RxCUI corresponding to *rxdrug_i*. |
| *tty_i* | RxNorm term type (TTY) for candidate *i* (e.g. IN, MIN). |
| *match_i* | Encoded match strategy used to derive candidate i (PERFECT, NORMALIZED_PERFECT, SUBSET, or MANUAL for manually edited candidates) |
| *rxcui_i_atc_j* | ATC code *j* associated with candidate *i*. |
| *keep* | List of integer values indicating the indices (*i*) of candidate mappings that are selected. The field is empty for automatic mapping exports and populated in restore-session outputs to preserve candidate selections required for reproducible session restoration. |
| *rxnorm_version* | Identifies the specific version of the RxNorm vocabulary used to generate associated medication mappings. |

In addition to drug-level candidate mappings, RxMap produces ATC classification sheets that explicitly encode ATC annotations for mapped RxNorm concepts. These outputs provide normalized therapeutic classification at multiple levels of the ATC hierarchy (ATC1–ATC4), enabling inspection of drug assignments at progressively finer therapeutic granularity. ATC outputs are generated automatically by propagating ATC codes from mapped RxNorm concepts and are included in both automatic and restore-session exports to provide explicit therapeutic classification and to capture ATC-level inclusion or decisions as they occur during manual review. Supplementary Table 4 shows the ATC classification schema at export.

**Supplementary Table 4.** ATC classification output schema for automatic and restore-session ATC exports.

| **Column** | **Description** |
| --- | --- |
| *Drug* | Original free-text medication string from which the ATC assignment was derived. |
| *rxdrug* | RxNorm drug concept used as the basis for ATC assignment. |
| *rxcui* | RxNorm Concept Unique Identifier (RxCUI) corresponding to *rxdrug*. |
| *atc_code* | Assigned ATC code. |
| *ATC1* | Human-readable description of the ATC anatomical main group. |
| *ATC2* | Human-readable description of the ATC therapeutic subgroup. |
| *ATC3* | Human-readable description of the ATC pharmacological subgroup. |
| *ATC4* | Human-readable description of the ATC chemical subgroup. |
| *keep* | Y/N indicator specifying whether the ATC assignment is kept. This field is empty in automatic ATC mapping outputs and populated in restore-session files to encode the retained ATC assignments used for deterministic session reconstruction. |
| *source* | Provenance indicator describing how the ATC assignment was generated: Auto (direct RxNorm annotation or strict ingredient-level validated inference), Partial (validated subset-level coverage when no exact multi-ingredient ATC exists), or Manual (introduced through manual curation outside the automated workflow). |
| *rxnorm_version* | Identifies the specific version of the RxNorm vocabulary used to generate associated ATC mappings. |

It should be noted that both drug mapping and ATC classification outputs include additional sheets suffixed with IN, which report exclusively ingredient-level (IN) mappings generated by decomposing all multi-ingredient (MIN) RxNorm concepts into their constituent ingredients. In contrast, non-suffixed sheets retain the original multi-ingredient concepts.

### Finalized (reviewed) drug-level mapping outputs

Finalized mapping sheets represent the authoritative, human-reviewed state of RxMap outputs. These sheets are structurally similar to automatic mappings but differ in two important respects: (i) only finalized drug and RxCUI assignments are retained, and (ii) intermediate decision flags are omitted. As a result, finalized mapping sheets are analysis-ready and do not encode review workflow mechanics.

## Provenance and auditability

RxMap implements provenance tracking by encoding metadata directly within its schemas. In automatic and restore-session outputs, columns such as source, *match_i*, *tty_i*, *rxnorm_guess*, and *llm_guess* document how each candidate mapping was generated. The *keep* flag column records explicit human acceptance or rejection decisions during review. Finalized outputs do not encode intermediate workflow metadata. Instead, auditability is ensured by automatic mappings and restore-session files, which record candidate generation, selection, and modification events and enable reconstruction of the full mapping history.

## Reproducibility considerations

Preprocessing, normalization, and ATC classification steps are fully deterministic given fixed inputs, configuration parameters, and reference resources. LLM-assisted candidate generation is likewise deterministic under fixed model configuration, as inference is performed with temperature set to zero and generated outputs are cached with associated provider and model identifiers. Restore-session exports preserve this determinism by enabling exact reconstruction of prior states.

# DATA AND GOLD-STANDARD MAPPINGS

RxMap was evaluated using medication data from two datasets: IPUM MEPS and a harmonized dataset denoted CHARTER/NNTC/HNRP. The first dataset was extracted from IPUMS Medical Expenditure Panel Survey (MEPS), focusing on the RXNAME field, which contains unedited, pharmacy-provided drug names reflecting the heterogeneous free-text strings encountered in real-world survey and dispensing data. After deduplication, the IPUMS dataset comprised 22,624 unique RXNAME strings. The second dataset, which we denote as CHARTER/NNTC/HNRP, was extracted from a harmonized longitudinal HIV dataset compiled from three studies spanning from 1999 to 2020: the CNS HIV Antiretroviral Therapy Effects Research (CHARTER), the National NeuroAIDS Tissue Consortium (NNTC), and HIV Neurobehavioral Research Program (HNRP). The data contains demographic information, HIV status, selected clinical measurements, and self-reported medications strings. After deduplication, the CHARTER/NNTC/HNRP dataset had 1363 unique drug name strings. Across both datasets, each entry was manually reviewed and mapped to establish a gold-standard RxCUI and its corresponding ingredient set. To support consistency and reduce potential bias, the annotations for the IPUMS data were cross-referenced against the RXDRGNAM field provided by IPUMS, which contains the generic drug name most commonly used by prescribing physicians. This field is assigned by IPUMS using the Multum Lexicon database and serves as an external reference for validation. However, discrepancies between RXDRGNAM and the correct map did occur, and such cases were manually reviewed and adjudicated to ensure accurate RxNorm mappings.
